# GWAS of agronomic traits in soybean collection included in breeding pool in Kazakhstan

**DOI:** 10.1186/s12870-017-1125-0

**Published:** 2017-11-14

**Authors:** Alibek Zatybekov, Saule Abugalieva, Svetlana Didorenko, Yelena Gerasimova, Ivan Sidorik, Shynar Anuarbek, Yerlan Turuspekov

**Affiliations:** 1 Institute of Plant Biology and Biotechnology, Almaty, Kazakhstan 050040; 2 Kazakh Research Institute of Agriculture, Almalybak vil., Almaty region Kazakhstan 040909; 3 East Kazakhstan Research Institute of Agriculture, Solnechnyi vil., Ust-Kamenogorsk region Kazakhstan 070518; 4 Kostanai Research Institute of Agriculture, Zarechnoe vil., Kostanai region Kazakhstan 111108

**Keywords:** Soybean, GWAS, Single nucleotide polymorphisms, Yield components

## Abstract

**Background:**

In recent years soybean is becoming one of the most important oilseed crops in Kazakhstan. Only within the last ten years (2006–2016), the area under soybean is expanded from 45 thousand hectares (ha) in 2006 to 120 thousand ha in 2016. The general trend of soybean expansion is from south-eastern to eastern and northern regions of the country, where average temperatures are lower and growing seasons are shorter. These new soybean growing territories were poorly examined in terms of general effects on productivity level among the diverse sample of soybean accessions. In this study, phenotypic data were collected in three separate regions of Kazakhstan and entire soybean sample was genotyped for identification of marker-trait associations (MTA).

**Results:**

In this study, the collection of 113 accessions representing five different regions of the World was planted in 2015–2016 in northern, eastern, and south-eastern regions of Kazakhstan. It was observed that North American accessions showed the highest yield in four out of six trials especially in Northern Kazakhstan in both years. The entire sample was genotyped with 6 K SNP Illumina array. 4442 SNPs found to be polymorphic and were used for whole genome genotyping purposes. Obtained SNP markers data and field data were used for GWAS (genome-wide association study). 30 SNPs appear to be very significant in 42 MTAs in six studied environments.

**Conclusions:**

The study confirms the efficiency of GWAS for the identification of molecular markers which tag important agronomic traits. Overall thirty SNP markers associated with time to flowering and maturation, plant height, number of fertile nodes, seeds per plant and yield were identified. Physical locations of 32 identified out of 42 total MTAs coincide well with positions of known analogous QTLs. This result indicates importance of revealed MTAs for soybean growing regions in Kazakhstan. Obtained results would serve as required prerequisite for forming and realization of specific breeding programs towards effective adaptation and increased productivity of soybean in three different regions of Kazakhstan.

**Electronic supplementary material:**

The online version of this article (10.1186/s12870-017-1125-0) contains supplementary material, which is available to authorized users.

## Background

Soybean is one of the most important oilseeds as well as protein source crop worldwide. In Kazakhstan, soybean planting area has been increased from 45 thousands hectares in 2006 to 120 thousands in 2016 [1]. This massive enlargement is under crop diversification policy adopted by KZ government with the final goal to reach up to 400 thousand hectares by 2020 occupied by commercial soybean annually. Designated areas of the expansion is land devoted to agriculture in south-eastern (SEK), eastern (EK) and northern (NK) regions of KZ [2, 3].

Soybean is a new crop for Kazakhstan. It dictates the necessity of preliminary studies such as evaluation of a number of diverse soybean varieties grown in the new environment and their potential use as an acceptor germplasm for the specific conventional breeding purposes. Recent findings regarding non-random associations between certain alleles of flowering genes and yield in soybean lines grown in these targeted regions [1] were a promising start in this direction. In this study, the time flowering span was assessed in all three regions in association with variation in *E* genes and yield performance. Specific allele combinations of the four *E* genes and respective optimal ranges of flowering and maturity time were identified for each experimental site [1]. However, more comprehensive research-based support is needed for successful development and implementation of specific conventional soybean breeding programs in KZ.

Several genomic oriented tools were generated in soybean community in recent years to facilitate breeding programs worldwide (soybase.org). The list of these genomic tools includes assembly of Williams 82 genomic sequence (http://www.soybase.org /SequenceIntro.php), Affymetrix SoyChip annotation [4], searching engines for *Glycine max* and *Glycine soja* sequences (http://plants.ensembl.org/Glycine_max/Info/Index), SoySNP50K iSelect BeanChip from Illumina [5], and etc. The 50,000 (50 K) SNP iSelect array was found to be particularly instrumental in genetic mapping of QTL (quantitative trait loci) for complex agronomic traits, including abiotic [6, 7] and biotic stress resistances [8] and seed quality [9, 10].

Genome-wide association studies (GWAS) is considered to be one of the most promising approaches in the identification of QTL of agronomic traits [11, 12]. In soybean GWAS experiments indicated a strong bias towards environmental factors in MTA discoveries [13–15]. For instance, obtained results from three different GWAS studies related to the identification of QTLs for yield performance in Canada [16], USA [13, 15], and Brazil [11] showed different responses and QTLs for yield components were identified in different parts of the genome. Presumably, in order to generate a reliable data for regionally running breeding projects, separate MTA experiments are required. The main objective of this study was to identify non-random MTAs in soybean field trials in three different environments in KZ. This is the first attempt based on association mapping approach for identification of soybean productivity with related QTLs in Kazakhstan.

## Results

### Phenotypic variation of the collection and GEI patterns

**Fig. 1:**
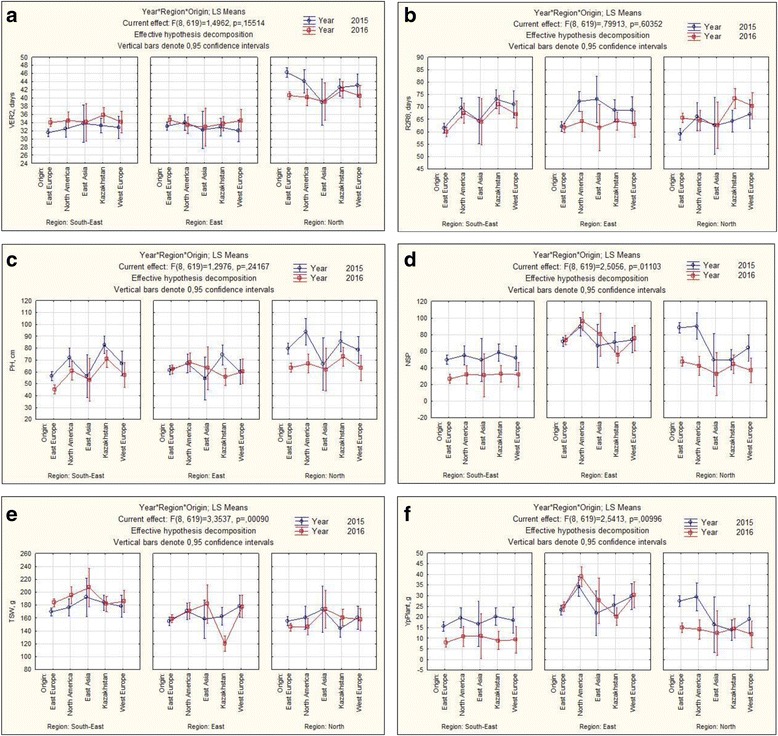
Results of comparative assessment by STATISTICA. **a** time to flowering (VER2) **b** seed maturation (R2R8), **c** plant height (PH), **d** number of seeds per plant (NSP), **e** thousand seed weight (TSW), **f** yield per plant (YP)

Overall highest average yield in the collection of 113 accessions over two years study was recorded in EK (26.53 + 1.09), followed by NK (18.19 + 0.72), and SEK (12.88 + 0.39). Comparative assessment of five groups of origin in the studied soybean collection in three regions during 2015–2016 has revealed sharp differences in time to flowering (VER2) and seed maturation (R2R8), plant height (PH), number of seeds per plant (NSP), thousand seed weight (TSW) and yield per plant (YP) (Fig. 1a-f).

Soybean varieties bred in North America showed highest yield potential in the majority of field trials. This trend was especially notable in North and East regions with the highest yield being observed in EK in 2015 (Fig. 1f). Pearson correlation for two years trials in South-Eastern and Eastern regions showed that Yield was highly dependable from all tested yield components (PH, NFN, NP, NSP and TSW). However, while both flowering and seed maturation time were significantly associated with Yield in the South-East, in Eastern region the Yield was highly correlated with time to seed maturation (*P* < 0.0001), but not with flowering time. Correlation results of two years in the Northern site were not identical, as flowering time (*P* < 0.001) and plant height (*P* < 0.02) were significantly related to the Yield in NK2015, but both traits were unrelated to the Yield in NK2016. Breeding origin of the collection exhibited a significant interaction with selected yield components in six spatiotemporal environments (O x R x Y) and place of growing (O x R) (Table 1).

**Table 1:** Four-way ANOVA by STATISTICA

Source	d.f.	VER2	R2R8	PH	NSP	TSW	YP
Accession	112	1.6*	2.9***	3.5***	1.9*	0.083 ns	1.8*
Origin	4	0.8 ns	23.9***	16.2***	6***	6.5***	7.9***
Region	2	104***	0.6 ns	11.5***	47***	25.5***	49.8***
Year	1	0.02 ns	0.97 ns	17.8***	29***	0.37 ns	16.2***
OxR	8	1.8 ns	1.9 ns	4.7***	4.9***	2.4*	5.5***
RxY	2	6.1**	6.4**	3.5*	9.5***	1.8 ns	7.3***
OxY	4	0.9 ns	2.4*	1.2 ns	0.7 ns	1.3 ns	0.3 ns
AxR	224	0.43 ns	0.55 ns	0.49 ns	1.01 ns	0.49 ns	1.7*
AxY	112	0.37 ns	0.64 ns	0.18 ns	0.56 ns	0.26 ns	0.51 ns
YxRxO	8	1.5 ns	0.8 ns	1.3 ns	2.5*	3.4***	2.5**
AxRxY	224	0.14 ns	0.69 ns	0.12 ns	0.98 ns	0.12 ns	0.92 ns
AxOxRxY	896	0.34 ns	0.45 ns	57.9***	1.18 ns	0.05 ns	1.07 ns

The F values are provided with significance level indicated by the asterisks

***P < 0.001, **P < 0.01, *P < 0.05, ns—not significant

**Fig. 2:**
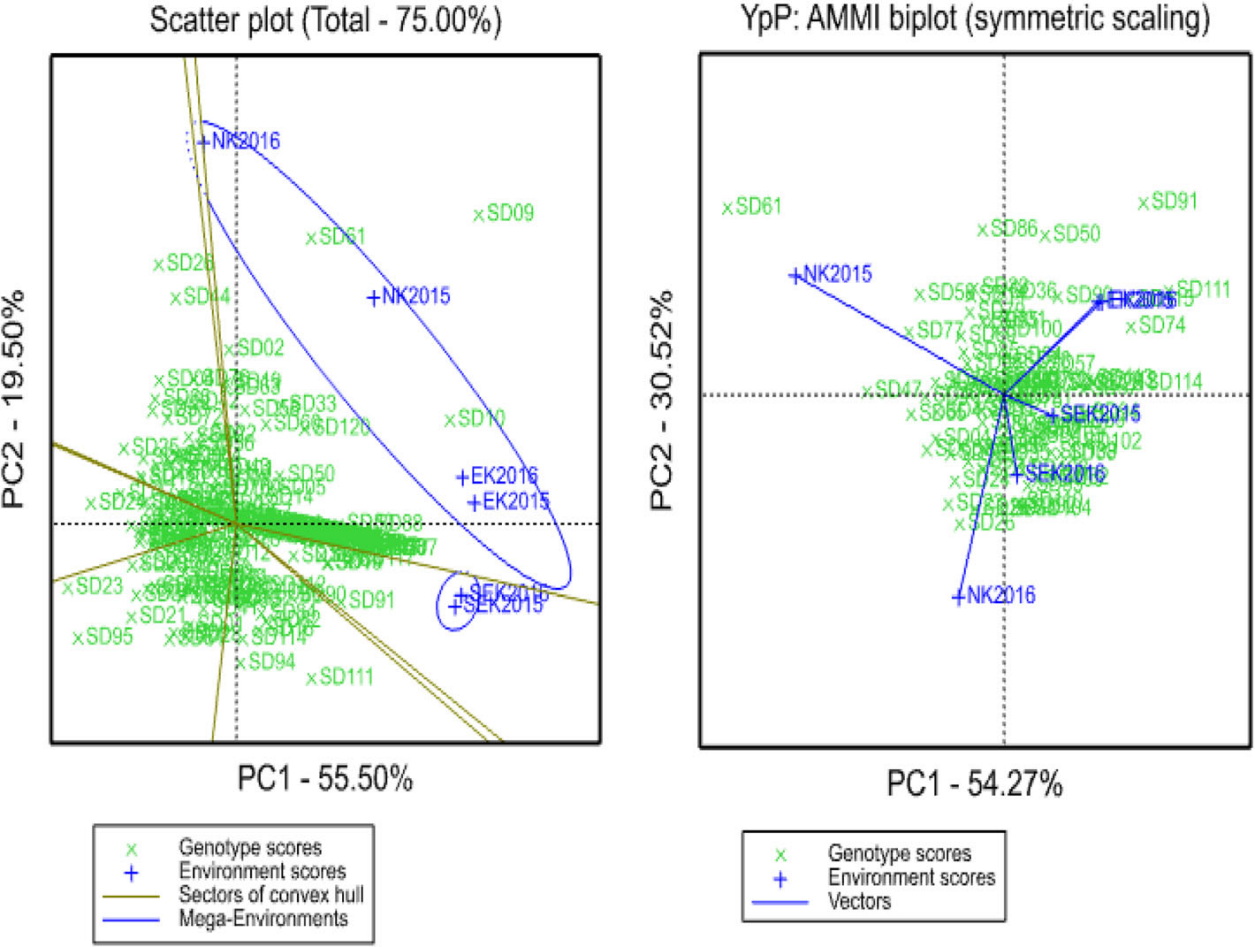
GGE and AMMI biplot graphic on genotype–environment interaction studies

Pearson’s correlation among six trials suggested that tests in NK unrelated to EK and SEK sites. This result was in well agreement with GGE biplot and AMMI results (Fig. 2). Particularly, AMMI symmetric scaling test as PC1 separated two NK sites from EK and SEK field studies (Fig. 2b). The ANOVA test for yield performance in three regions suggested that Environment (E) significantly influenced the genotype x environment interaction, where E contributed 81.9%, while G and GE provided only 18.1% together.

### Genetic variation in the soybean collection based on SNP markers

**Fig. 3:**
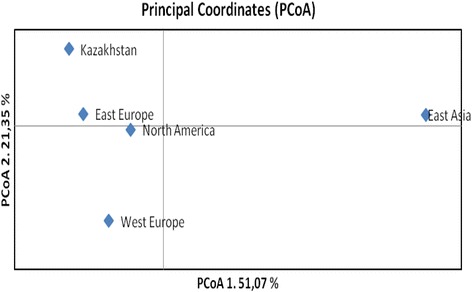
Principal coordinate analysis for 113 soybean accessions separated into 5 groups according to their breeding origin (Additional file 6) based on 4442 SNPs

Genotyping soybean collection using Illumina iSelect SNP array revealed 4442 polymorphic SNPs (74.03% success) with 77.98% variants being transitions and 22.01% transversions. The principal coordinate analysis (PCoA) allowed the group of all 113 accessions based on their breeding origin. The accessions were split into 5 geographically distinct groups. The number of accessions within each group was uneven. The East Asian group was represented by 3 samples only and East European group was represented by 67 accessions (Table 2). The PCoA1 graph reveals that genotypes from East Asia are genetically more distant from other four groups (Fig. 3). The PCoA2 is effectively separated remaining four groups, as East European and North American accessions appeared to be most close groups, and Kazakhstan accessions have the closest similarity to varieties from East Europe (Fig. 3).

**Table 2:** Mean genetic diversity indexes in five soybean groups based on 4442 SNPs

Population	East Europe	West Europe	East Asia	North America	Kazakhstan
N^a^	67	9	3	16	18
Ne^b^	1.82 ± 0.007	1.69 ± 0.006	1.40 ± 0.006	1.61 ± 0.005	1.62 ± 0.005
I^c^	0.67 ± 0.004	0.57 ± 0.004	0.32 ± 0.004	0.51 ± 0.003	0.53 ± 0.004
uh^d^	0.40 ± 0.003	0.40 ± 0.003	0.33 ± 0.005	0.36 ± 0.002	0.36 ± 0.003

^a^Number of accessions

^b^Number of effective alleles

^c^Shannon index

^d^Unbiased Nei’s diversity index

### Association mapping

The genotyping data consisted of 4442 SNPs spanned on 20 chromosomes with the average length of 47.4 Mb and the average number of SNPs per chromosomes of 222.1. The number of markers per chromosome varied from 163 in Gm11 to 286 in Gm13, and length of the chromosome ranged from 37.3 Mb in Gm16 to 62.2 Mb in Gm18. The average density of the SNP map was one marker per every 213 Kb. The LD decay curve (Additional file 1) at the threshold *r* ^*2*^ = 0.1 was 20 Kbp. The application of the MLM (mixed linear model) with K plus Q matrices in the GWAS resulted in identification of 46 SNPs for 64 MTAs (Additional file 2). Obtained results were further statistically validated using the *t-test* for identification of false positive MTAs. After the application of the t-test, 30 SNPs revealed for identification of 42 true MTAs in six studied environments (Table 3). The revealed 42 MTAs were associated with time to flowering (VER2) and maturation (VER8), plant height (PH), height of first branch (HFB), number of fertile nodes (NFN), number of seeds per plant (NSP), thousand seed weight (TSW), and weight per plant (YPP) were selected for further analyses (Table 3). The results suggested that 22 MTAs were significant with traits related to adaptation related traits, and 20 MTAs were related to the yield components. Only four MTAs, found to be significant in two or more regions, were identified and all of them were related to plant growth traits. In terms of regional distribution, 9 MTAs were identified for eastern region and 33 MTAs were in both south-eastern and northern regions (Table 3).

**Table 3:** The list of MTAs and SNP markers significant with main agronomic traits

Traits	Chr.	Position	*P* value	R^2^(%)	MAF	Allele	Effect
VER2	06	20,370,075	5.8011E-5	24.6	0.179	C/T	5.77
	19	49,964,637	7.2399E-6	21.2	0.152	C/T	5.73
R2R4	2	45,940,601	2.4647E-5	24.0	0.232	C/T	−10.18
	10	3,066,211	1.2186E-5	23.5	0.268	C/T	−1.84
	17	14,418,215	5.4006E-5	18.9	0.116	A/G	−5.13
	20	3,020,597	1.0172E-5	23.3	0.313	C/T	−3.43
	20	14,721,991	4.6494E-6	20.5	0.156	A/G	12.37
	20	23,536,158	2.6634E-5	17.1	0.344	A/G	−7.95
R2R8	5	8,597,246	4.0476E-5	14.7	0.063	C/T	17.73
	20	3,020,597	1.0172E-5	23.3	0.313	C/T	−3.43
	20	8,185,857	4.1578E-6	13.9	0.085	C/T	−6.03
R4R8	5	8,597,246	4.0476E-5	14.7	0.063	C/T	17.73
	14	9,803,364	8.8788E-5	19.5	0.219	C/T	−12.50
	14	28,158,698	1.9669E-5	18.4	0.290	C/T	4.85
	19	27,283,886	3.1538E-5	21.2	0.321	C/T	4.92
VER8	10	48,586,134	5.5824E-5	24.9	0.259	A/C	3.32
	14	7,151,265	8.5988E-5	17.3	0.446	A/G	−10.63
	19	48,168,077	1.0025E-5	30.7	0.201	A/G	−6.81
	20	3,020,597	1.0172E-5	23.3	0.313	C/T	−3.43
	20	8,185,857	4.1578E-6	13.9	0.085	C/T	−6.03
	20	14,721,991	4.6494E-6	20.5	0.156	A/G	12.37
	20	23,536,158	2.6634E-5	17.1	0.344	A/G	−7.95
PH	9	42,241,644	4.7641E-5	15.9	0.487	A/G	−14.94
	20	8,185,857	4.1578E-6	13.9	0.085	C/T	−6.03
HFB	9	42,578,079	4.1561E-5	17.2	0.112	A/G	4.20
	20	40,765,691	3.8709E-5	18.5	0.299	A/G	5.46
NFN	14	9,803,364	8.8788E-5	19.5	0.219	C/T	−12.50
	19	30,103,637	8.2608E-5	17.3	0.317	G/T	−5.17
NSP	8	14,431,777	1.6689E-5	31.0	0.152	A/C	5.04
	10	981,062	1.7273E-5	30.5	0.156	A/G	−7.73
	20	8,185,857	4.1578E-6	13.9	0.085	C/T	−6.03
	20	30,417,244	4.7329E-5	15.7	0.094	C/T	−13.06
TSW	2	12,244,605	6.6226E-5	22.9	0.496	A/G	−31.82
	4	516,796	8.8647E-5	21.7	0.335	A/G	−33.08
	5	3,859,212	1.1104E-5	24.8	0.357	C/T	−32.22
	7	16,031,010	6.2361E-5	18.8	0.161	C/T	−22.28
	17	10,106,704	2.7099E-5	24.7	0.107	C/T	−49.48
	20	14,721,991	4.6494E-6	20.5	0.156	A/G	12.37
YP	14	27,937,142	3.9461E-5	15.4	0.094	C/T	−4.66
	17	14,418,215	5.4006E-5	18.9	0.116	A/G	−5.13
	20	8,185,857	4.1578E-6	13.9	0.085	C/T	−6.03
	20	30,417,244	4.7329E-5	15.7	0.094	C/T	−13.06

The analysis of genome physical locations of associated SNP markers revealed that 10 out of 30 totally identified were part of CDS (coding DNA sequence) and remaining 20 SNPs were located in inter genic regions (Additional file 3). Each SNP in inter genic position was considered for potential functional annotation based on the actual proximity of nearby located genes. In addition, the physical position of each critical SNP marker was superimposed on positions of known QTLs (https://soybase.org/search/qtllist_by_symbol.php). Interesting to note that 32 out of 42 MTAs goes exactly where analogous QTL were positioned in soybean genome (Additional file 4). For instance, two QTLs for seed weight were in the same positions where for two out of three MTAs for TSW were identified, four QTLs for seed weight were found for four MTAs for NSP, internode length QTL was found for the same position of the MTA for PH, and etc. (Table 4). According to Soybase there were 8 SNPs as part of 9 MTAs for which no QTLs were found, although 4 out of 8 of these SNPs were located in CDS regions.

## Discussion

113 soybean accessions from 5 different breeding origins were compared for yield performance in three soybean growing regions of Kazakhstan. North American accessions demonstrated the highest yield in the majority of trials (Fig. 1), supporting superior adaptability of this genetic material to the KZ environment. It is interesting to note that the pedigree of a few local high-performance cultivars also included in this study are heavily based on the US bred soybean background. On the other hand, principal coordinate analysis (PCA) showed that the most of local varieties genetically closer to East European germplasm, although North American and East European accessions were not far apart from each other (Fig. 3).

The size and level of genetic variation in studied genetic panels appear to be critical for the positive outcome of GWAS based projects. It was demonstrated that experiments with sample size less than 384 accessions [17] and large LD blocks [18, 19] may lead to the identification of false positive associations. On the other hand, in the study by Turner et al. 2016, there was shown that smaller panels may allow detection of false negative associations that would not have been detected in the larger panels [20]. These study results on relatively small soybean sample size (*n* = 113) in fact confirm the above mentioned findings. Initially, 64 MTAs were detected using TASSEL MLM model with the application of K + Q matrices. However, further validation of the revealed associations using *t-test* suggested that only 42 of them are presumably true associations. GWAS led to the identification of 30 SNP markers involved in 42 confirmed MTAs. Comparison of physical positions of identified SNPs with positions of previously mapped QTLs (https://soybase.org/search/qtllist_by_symbol.php) showed that 32 out of 42 identified MTAs have been found to be at the same locations as known QTLs. Remaining 10 MTAs identified in this study might be denoted as presumably novel QTLs. Also, a high percentage of positive matches regarding physical locations of identified SNPs and known QTLs support GWAS as a useful research tool for the search of non-random MTAs in soybean in Kazakhstan.

The other causative factor perhaps relevant to the identification of novel QTLs in this study is the impact of specific environmental conditions listed in Additional file 5. Here significant environmental influence (81.9%) on yield performance was detected by ANOVA in the genotype x environment interaction (GEI) assessment. Four MTAs were significant in two or more field trials, and this can be apparently explained by large environmental effect causing a relatively low stability of the identified loci.

In this study, 24 MTAs related to plant adaptation traits including plant height were identified. For the length of flowering time, two MTAs were revealed on chromosomes Gm06 and Gm19 (Table 3). The Gm06_20,370,075 was located in the close proximity to the *GmPhyA1* gene, which was annotated as a phytochrome receptor corresponding to the flowering E1 locus [21]. Gm19_49,964,637 has located approximately 2.5 Mb from the *GmPhyA3* gene, another phytochrome receptor corresponding to the maturity E3 locus [22]. Four identified SNPs, including those on chromosomes Gm10 and Gm19, were associated with maturity time, and their map locations coincided with the genetic positions of *E2* and *E3* maturity loci [21, 22]. Gm10_48,586,134 in chromosome Gm10 was approximately 3.9 Mb apart from *GmGIa* (Glyma10g36600), a gene that involved regulation of soybean maturity and flowering time [23]. The Gm19_48,168,077 in chromosome Gm19, as in case of flowering MTA, was located approximately 1.5 Mb from the *GmPhyA3* gene (Glyma19g41210). It is interesting to note, that location of another SNPs Gm07_10009107 was in proximity to the *Phytochrome B* (Glyma07g11790) gene, which commonly encodes inhibitory effect on plant germination [24]. SNP location of the Gm09_42,241,644, which is related to the MTA for PH, was coincided with the physical position of previously mapped QTL for internode length (soybase.org) (Additional file 3).

The second group of identified MTAs belongs to yield component traits, which includes NFN, NSP, TSW, and YPP. In total, GWAS allowed the identification of 20 MTAs for yield components spread on ten different chromosomes (Table 3).

## Conclusions

Overall thirty SNP markers associated with time to flowering and maturation, plant height, number of fertile nodes, seeds per plant and yield were identified. Physical locations of 32 identified out of 42 total MTAs coincide well with positions of known analogous QTLs (www.soybase.org). This result indicates importance of revealed MTAs for soybean growing regions in Kazakhstan. The other 10 MTAs might lay claim to some novelty until additional proof is obtained. Obtained results would serve as required prerequisite for forming and realization of specific breeding programs towards effective adaptation and increased productivity of soybean in three different regions of Kazakhstan.

## Methods

### Plant material

Soybean sample consisted of 113 accessions, including 18 released cultivars and prospective breeding lines from Kazakhstan (Additional file 6) were used in this study [1]. Plant research material represents 12 countries from 5 geographic regions, including Western and Eastern Europe, North America, East Asia, and Kazakhstan. All 113 accessions were grown in 2015–2016 in three randomized replications in three agricultural regions in KZ. Specifically research plots were kindly provided by crop breeding stations located in South-East, East, and North regions of Kazakhstan. Besides distinctive environmental differences between these regions farmland in SEK represented by irrigated agriculture, unlike those in EK and NK stations. Exact locations, respective meteorological data and field conditions are shown in Additional file 5. Plants were grown in 1 m long rows with 30 cm distance between adjacent rows and 5 cm space between plants within rows.

### Phenotypic analysis study

Statistical analyses of obtained data were calculated by using GraphPad Prism 7.0 (https://www.graphpad.com/scientific-software/prism) and STATISTIKA 13.2 (http://software.dell.com/products/statistica) computer programs. Genotype-environment interactions, including AMMI (Additive Main Multiplicative Interaction) and GGE Biplot methods, were analyzed by GenStat software package (https://www.vsni.co.uk/software/genstat/). The symmetric scaling option of both methods and available field data for all three sites were used in estimations. The key property of a GGE biplot is that it is based on Tester-Centered data, whereby the tester (environment) main effects (E) are removed, and the entry main effect (G) and the entry by tester interaction (GE) are retained and combined.

### DNA genotyping and genetic variation study

DNA samples were extracted and purified from single seeds of individual cultivars using commercial kits (Qiagene, CA, USA). The DNA concentration for each sample was adjusted to 50 ng/μl. All samples were genotyped using the soybean 5403 Illumina iSelect SNP array [5] at the Traitgenetics GmbH (Gatersleben, Germany). The Illumina Infinium procedure was performed according to the manufacturer’s protocol. SNP genotype analysis was carried out using the Illumina Genome Studio software (GS V2011.1). Population genetic analysis and principal coordinate analysis were performed using GenAlEx 6.5 [25].

### Association mapping study

The SNP dataset was filtered using a 10% cutoff for missing data and markers with minor allele frequency ≥ 0.10 were considered for GWAS. Numbers of hypothetical groups ranging from k = 1 to 10 were assessed using 50,000 burn-in iterations followed by 100,000 recorded Markov-Chain iterations. To estimate the sampling variance of population structure inference, five independent runs were carried out for each *k*. The output from STRUCTURE was analyzed for delta K value (ΔK) in STRUCTURE HARVESTER [26]. On the basis of the final *k* values, Q-matrix for three identified clusters was developed. GWAS for quantitative traits of plant growth and yield components were studied using 4442 filtered SNPs against minor alleles. Genome-wide association mapping based on the MLM + Q + K model was conducted using TASSEL 5 [27].

## Additional files

Additional file 1: Linkage disequilibrium decay in pairwise analysis. (PDF 8 kb)

Additional file 2: The list of total MTAs identified by Tassell 5.0 package. (PDF 184 kb)

Additional file 3: Physical positions of identified SNP in soybean genome. (PDF 18 kb)

Additional file 4: Comparison of SNP in identified MTAs in this study and QTL locations in soybean database*. (PDF 160 kb)

Additional file 5: Locations and meteorological data for three experimental sites for soybean field trials. (PDF 219 kb)

Additional file 6: The list of soybean accessions and their origin. (PDF 405 kb)
