# Supplementary figures and images for: GWAS of agronomic traits in soybean collection included in breeding pool in Kazakhstan
Source: BMC Plant Biol. 2017 Nov 14;17(Suppl 1):179. doi: 10.1186/s12870-017-1125-0 (PMC5688460; doi:10.1186/s12870-017-1125-0)

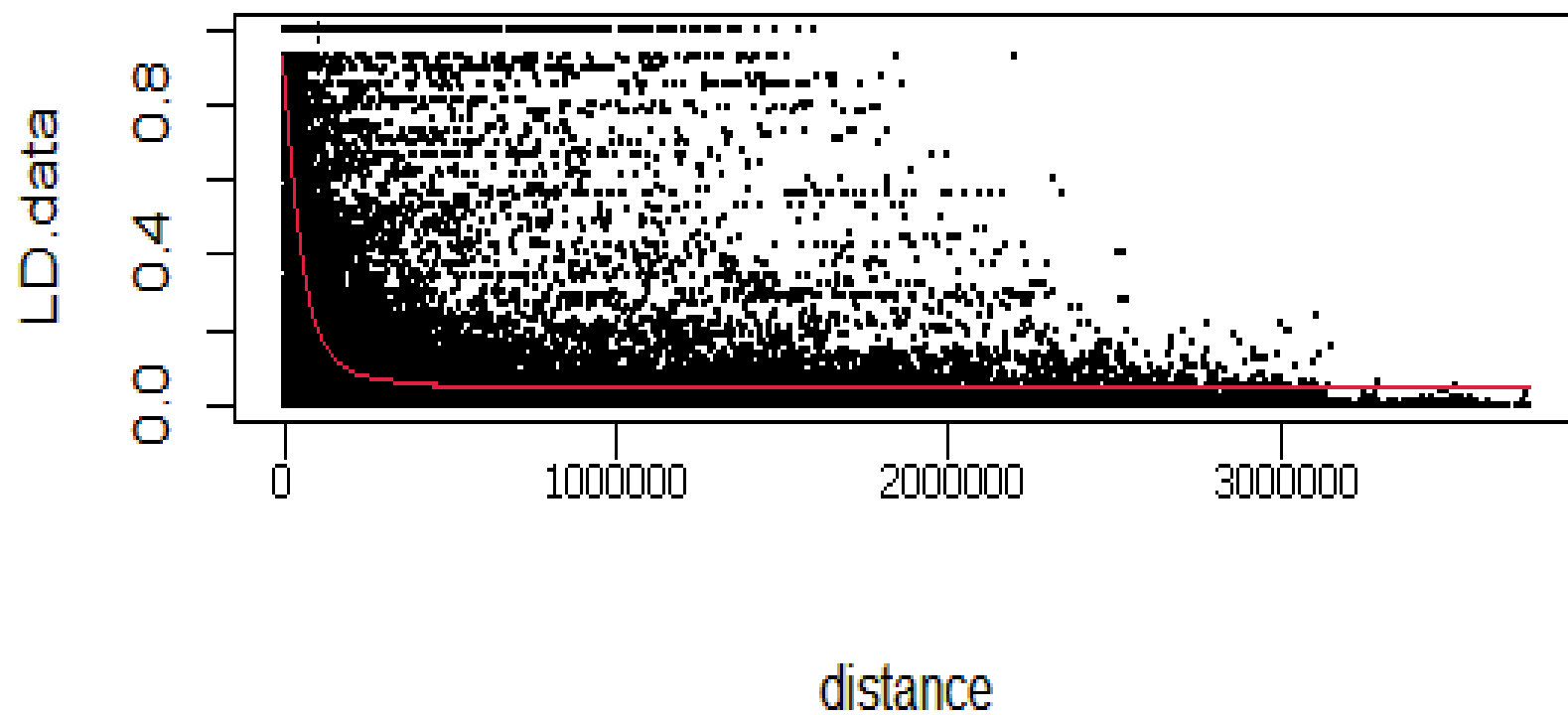

Supplement: Supplementary file 1 — Linkage disequilibrium decay in pairwise analysis. (PDF 8 kb) [file 12870_2017_1125_MOESM1_ESM.pdf]
