# Supplementary material for: GWAS of agronomic traits in soybean collection included in breeding pool in Kazakhstan
Source: BMC Plant Biol. 2017 Nov 14;17(Suppl 1):179. doi: 10.1186/s12870-017-1125-0 (PMC5688460; doi:10.1186/s12870-017-1125-0)
Supplement: Supplementary file 2 — The list of total MTAs identified by Tassell 5.0 package. (PDF 184 kb) [file 12870_2017_1125_MOESM2_ESM.pdf]

Traits	Chr.	Position	*P*-value	R2(%)	MAF	Allele	Effect
VER2	6	19540686	3.0896E-5	20.1	0.393	C/T	4.37
	6	21363243	5.8011E-5	24.6	0.179	A/G	-5.77
	17	2899237	1.1343E-5	25.4	0.165	A/G	1.61
	19	49964637	7.2399E-6	21.2	0.152	C/T	5.73
	20	2804571	7.39203E-6	25.3	0.129	C/T	-5.38
R2R4	2	45940601	2.4647E-5	24	0.232	C/T	-10.18
	10	3066211	1.2186E-5	23.5	0.268	C/T	-1.84
	15	14712034	2.8393E-5	15.6	0.223	G/T	-5.46
	17	14418215	5.4006E-5	18.9	0.116	A/G	-5.13
	20	3020597	1.0172E-5	23.3	0.313	C/T	-3.43
	20	14721991	4.6494E-6	20.5	0.156	A/G	12.37
	20	18829763	2.1263E-5	20.6	0.170	A/G	10.57
	20	23536158	2.6634E-5	17.1	0.344	A/G	-7.95
R2R8	5	8597246	4.0476E-5	14.7	0.063	C/T	17.73
	8	25219400	5.0843E-5	13.9	0.143	A/G	13.24
	8	34085111	5.0843E-5	15.8	0.143	C/T	13.24
	16	32017661	5.1119E-5	15.2	0.321	A/C	11.58
	20	3020597	1.0172E-5	23.3	0.313	C/T	-3.43
	20	8185857	4.1578E-6	13.9	0.085	C/T	-6.03
R4R8	5	8597246	4.0476E-5	14.7	0.063	C/T	17.73
	11	3641716	8.8828E-5	16.2	0.255	A/C	-5.48
	14	9803364	8.8788E-5	19.5	0.317	G/T	-5.17
	14	19373649	8.0379E-5	15.1	0.362	A/G	-4.56
	14	28158698	1.9669E-5	18.4	0.290	C/T	4.85
	19	14896890	9.8268E-5	18.3	0.348	A/G	-4.4
	19	27283886	3.1538E-5	21.2	0.321	C/T	4.92
VER8	1	4120447	9.8173E-5	15.6	0.129	C/T	-11.66
	3	1374930	9.516E-5	16.7	0.438	G/T	-10.39
	7	10009107	5.6685E-5	17.1	0.321	A/G	12.78
	8	25219400	5.0843E-5	13.9	0.143	A/G	13.24
	8	34085111	5.0843E-5	15.8	0.143	C/T	13.24
	10	48586134	5.5824E-5	24.9	0.259	A/C	3.32
	14	7151265	8.5988E-5	17.3	0.446	A/G	-10.63

VER8	16	32017661	5.1119E-5	15.2	0.321	A/C	11.58
	19	48168077	1.0025E-5	30.7	0.201	A/G	-6.81
	20	3020597	1.0172E-5	23.3	0.313	C/T	-3.43
	20	8185857	4.1578E-6	13.9	0.085	C/T	-6.03
	20	14721991	4.6494E-6	20.5	0.156	A/G	12.37
	20	23536158	2.6634E-5	17.1	0.344	A/G	-7.95
PH	9	42241644	4.7641E-5	15.9	0.487	A/G	-14.94
	20	8185857	4.1578E-6	13.9	0.085	C/T	-6.03
HFB	9	42578079	4.1561E-5	17.2	0.112	A/G	4.20
	20	40765691	3.8709E-5	18.5	0.299	A/G	5.46
NFN	14	9803364	8.8788E-5	19.5	0.317	G/T	-5.17
	19	30103637	8.2608E-5	17.3	0.219	C/T	-12.50
	16	32017661	5.1119E-5	15.2	0.321	A/C	11.58
NSP	8	14431777	1.6689E-5	31	0.152	A/C	5.04
	10	1051336	4.8032E-5	26.9	0.174	A/G	-2.44
	10	711291	7.9181E-5	25.2	0.210	A/G	13.50
	10	981062	1.7273E-5	30.5	0.156	A/G	-7.73
	16	32017661	5.1119E-5	15.2	0.321	A/C	11.58
	20	8185857	4.1578E-6	13.9	0.085	C/T	-6.03
	20	30417244	4.7329E-5	15.7	0.094	C/T	-13.06
TSW	2	12244605	6.6226E-5	22.9	0.496	A/G	-31.82
	4	516796	8.8647E-5	21.7	0.335	A/G	-33.08
	5	3859212	1.1104E-5	24.8	0.357	C/T	-32.22
	7	16031010	6.2361E-5	18.8	0.161	C/T	-22.28
	17	10106704	2.7099E-5	24.7	0.107	C/T	-49.48
	20	14721991	4.6494E-6	20.5	0.156	A/G	12.37
YP	14	27937142	3.9461E-5	15.4	0.094	C/T	-4.66
	17	14418215	5.4006E-5	18.9	0.116	A/G	-5.13
	20	8185857	4.1578E-6	13.9	0.085	C/T	-6.03
	20	30417244	4.7329E-5	15.7	0.094	C/T	-13.06
YPM	16	35643452	7.5376E-5	17.9	0.402	C/T	-44.34
