# Supplementary material for: GWAS of agronomic traits in soybean collection included in breeding pool in Kazakhstan
Source: BMC Plant Biol. 2017 Nov 14;17(Suppl 1):179. doi: 10.1186/s12870-017-1125-0 (PMC5688460; doi:10.1186/s12870-017-1125-0)
Supplement: Supplementary file 3 — Physical positions of identified SNP in soybean genome. (PDF 18 kb) [file 12870_2017_1125_MOESM3_ESM.pdf]

SNP ID	Type	Candidate gene	Annotation
Gm02_12244605	intron	Glyma02g13910	GH3 auxin-responsive promoter
Gm02_45940601	CDS	Glyma02g40700	Amine oxidase
Gm04_516796	CDS	Glyma04g00925	Unknown protein
Gm05_3859212	CDS	Glyma05g04690	Zinc finger protein
Gm05_8597246	intron	Glyma05g08700	Miro-like protein
Gm06_20370075	intron	Glyma06g23262	Kinesin motor domain
Gm07_16031010	intron	Glyma07g16320	Short-chain dehydrogenases/reductase
Gm08_14431777	CDS	Glyma08g19120	K+ potassium transporter
Gm09_42241644	intron	Glyma09g36510	Rna-binding protein related
Gm09_42578079	intron	Glyma09g37000	Xenotropic and polytropic murine leukemia virus receptor
Gm10_981062	CDS	Glyma10g01330	Myb-like DNA-binding domain
Gm10_3066211	CDS	Glyma10g04060	CGI-141-related/lipase containing protein
Gm10_48586134	CDS	Glyma10g41540	B-box zinc finger
Gm14_7151265	CDS	Glyma14g09150	Fms interacting protein
Gm14_9803364	intron	Glyma14g11320	Spermidine synthase
Gm14_27937142	intron	Glyma14g23545	Chromosome transmission fidelity factor 18
Gm14_28158698	intron	Glyma14g23754	Unknown protein
Gm16_35643452	intron	Glyma16g32500	Unknown protein
Gm17_10106704	intron	Glyma17g13210	Serine-threonine protein kinase, plant-type
Gm17_14418215	intron	Glyma17g17540	Protein of unknown function DUF260
Gm19_27283886	CDS	Glyma19g22490	Amp dependent ligase/synthetase
Gm19_30103637	intron	Glyma19g24520	Amino acid transporters
Gm19_48168077	CDS	Glyma19g42100	Ring finger protein 24-related
Gm19_49964637	intron	Glyma19g44580	AP2 domain
Gm20_3020597	intron	Glyma20g03310	Unknown protein
Gm20_8185857	intron	GLyma20g06271	Unknown protein
Gm20_14721991	intron	Glyma20g10600	Camp-response element binding protein-related
Gm20_23536158	intron	Glyma20g16793	Protein of unknown function (DUF 659)
Gm20_30417244	intron	Glyma20g21261	FAR1-related sequence 7
Gm20_40765691	intron	Glyma20g32120	Transferase family
