# Supplementary material for: GWAS of agronomic traits in soybean collection included in breeding pool in Kazakhstan
Source: BMC Plant Biol. 2017 Nov 14;17(Suppl 1):179. doi: 10.1186/s12870-017-1125-0 (PMC5688460; doi:10.1186/s12870-017-1125-0)
Supplement: Supplementary file 4 — Comparison of SNP in identified MTAs in this study and QTL locations in soybean database*. (PDF 160 kb) [file 12870_2017_1125_MOESM4_ESM.pdf]

Traits	Position	*P* value	Known QTLs*
VER2	Gm06_20370075	5.8011E-5	Total growth duration 1-4, Vegetative period 1-1, First flower 23-1, 22-2, 3-1, 9-1, 20-1, 10-1,
	Gm19_49964637	7.2399E-6	First flower 20-2, 16-4, 5-3, 8-3
R2R4	Gm02_45940601	2.4647E-5	
	Gm10_3066211	1.2186E-5	Row spacing response 1-2, 2-2
	Gm17_14418215	5.4006E-5	Reproductive period 1-7
	Gm20_3020597	1.0172E-5	Pod maturity 24-5, cqR8 Full maturity-001
	Gm20_14721991	4.6494E-6	Pod maturity 24-5, 11-1, cqR8 Full maturity-001
	Gm20_23536158	2.6634E-5	Pod maturity 24-5, 11-1, cqR8 Full maturity-001
R2R8	Gm05_8597246	4.0476E-5	
	Gm20_3020597	1.0172E-5	Pod maturity 24-5, cqR8 Full maturity-001
	Gm20_8185857	4.1578E-6	Pod maturity 24-5, 11-1, cqR8 Full maturity-001
R4R8	Gm05_8597246	4.0476E-5	
	Gm14_9803364	8.8788E-5	Seed weight 13-2, 36-14, 23-1
	Gm14_28158698	1.9669E-5	Reproductive period 1-6, Pod maturity 24-11, 19-4, 24-1, 27-3, 29-3
	Gm19_27283886	3.1538E-5	
VER8	Gm10_48586134	5.5824E-5	Pod maturity 13-8
	Gm14_7151265	8.5988E-5	
	Gm19_48168077	1.0025E-5	Reproductive stage length 3-2, 3-3, 7-4, Pod maturity 9-2, 9-3
	Gm20_3020597	1.0172E-5	Pod maturity 24-5, cqR8 Full maturity-001
	Gm20_8185857	4.1578E-6	Pod maturity 24-5, 11-1, cqR8 Full maturity-001
	Gm20_14721991	4.6494E-6	Pod maturity 24-5, 11-1, cqR8 Full maturity-001
	Gm20_23536158	2.6634E-5	Pod maturity 24-5, 11-1, cqR8 Full maturity-001
PH	Gm09_42241644	4.7641E-5	Internode length 1-6
	Gm20_8185857	4.1578E-6	Seed yield 10-1, 9-1, Seed weight 34-5, 8-1, 35-5, cqSeed weight-003,001
HFB	Gm09_42578079	4.1561E-5	Internode length 1-6
	Gm20_40765691	3.8709E-5	
NFN	Gm14_9803364	8.8788E-5	Seed weight 13-2, 36-14, 23-1
	Gm19_30103637	8.2608E-5	

NSP	Gm08_14431777	1.6689E-5	Seed weight 49-1, 35-1, 34-13
	Gm10_981062	1.7273E-5	Seed weight 12-6, Seed yield 15-2, 22-19
	Gm20_8185857	4.1578E-6	Seed yield 10-1, 9-1, Seed weight 34-5, 8-1, 35-5, cqSeed weight-003,001
	Gm20_30417244	4.7329E-5	Seed weight 34-5, 35-5, 9-1, cqSeed weight-003,001, Seed yield 14-1
TSW	Gm02_12244605	6.6226E-5	Seed weight 49-8
	Gm04_516796	8.8647E-5	Seed weight per plant 6-2
	Gm05_3859212	1.1104E-5	
	Gm07_16031010	6.2361E-5	
	Gm17_10106704	2.7099E-5	Seed weight 13-5, 47-2, 49-10
	Gm20_14721991	4.6494E-6	Seed yield 10-1, 9-1, Seed weight 34-5, 8-1, 35-5, cqSeed weight-003,001
YP	Gm14_27937142	3.9461E-5	Seed weight 13-2, Seed yield 31-1, 23-10, 32-3, 23-11, Seed yield to plant height related 3-4
	Gm17_14418215	5.4006E-5	Reproductive period 1-7, Seed weight 47-2, 49-10
	Gm20_8185857	4.1578E-6	Seed yield 10-1, 9-1, Seed weight 34-5, 8-1, 35-5, cqSeed weight-003,001
	Gm20_30417244	4.7329E-5	Seed weight 34-5, 35-5, 9-1, cqSeed weight-003,001, Seed yield 14-1

* - Based on the QTL list on SoyBase (https://soybase.org/search/qtllist_by_symbol.php)
