# Supplementary material for: GWAS of agronomic traits in soybean collection included in breeding pool in Kazakhstan
Source: BMC Plant Biol. 2017 Nov 14;17(Suppl 1):179. doi: 10.1186/s12870-017-1125-0 (PMC5688460; doi:10.1186/s12870-017-1125-0)
Supplement: Supplementary file 5 — Locations and meteorological data for three experimental sites for soybean field trials. (PDF 219 kb) [file 12870_2017_1125_MOESM5_ESM.pdf]

Index	South-east Kazakhstan		East Kazakhstan		North Kazakhstan	
	2015	2016	2015	2016	2015	2016
Average rainfall,mm	627,45		472,25		419,5	
Annual rainfall,mm	673,90	581,00	478	466,5	412	427
Veg,period rainfall	208,30	176,80	180,2	196	147,40	210,3
Mean T°C	23,01	21,64	18,7	23,59	17,4	23,9
Max T°C	31,70	28,50	29,4	32	27,3	35
Min T°C	13,30	9,60	4,2	9	1,6	11
Soil type	Light chestnut (humus 2.0-2.5%)		Chestnut soil (humus 1.3-2.9%)		Black soil (humus 4.5-5%)	
Laititude	43°21′		49°57′		53°45′	
Longitude	76°53′		82°37′		62°03′	
Elavation above sea level	740		287		189	
Date of sowing	4 May	20 Apr	13 May	16 May	23 May	17 May
Date of harwesting	7 Sep	18 Aug	25 Sep	18 Sept	12 Oct	2 Oct
