# Supplementary material for: GWAS of agronomic traits in soybean collection included in breeding pool in Kazakhstan
Source: BMC Plant Biol. 2017 Nov 14;17(Suppl 1):179. doi: 10.1186/s12870-017-1125-0 (PMC5688460; doi:10.1186/s12870-017-1125-0)
Supplement: Supplementary file 6 — The list of soybean accessions and their origin. (PDF 405 kb) [file 12870_2017_1125_MOESM6_ESM.pdf]

Accessions ID	Origin group	Origin Country	Accessions ID	Origin group	Origin Country
Yaselda	East Europe	Belarus	Eldorado	East Europe	Russia
Pripyat	East Europe	Belarus	Garmoniya	East Europe	Russia
OAC Vision	North America	Canada	Romantika	East Europe	Russia
Maplepresto	North America	Canada	Belgorodskaya6	East Europe	Russia
Emerson	North America	Canada	Veidelevskaya17	East Europe	Russia
P-73-3	North America	Canada	Yantarnaya	East Europe	Russia
Supra	North America	Canada	Altom	East Europe	Russia
Maple Ridge	North America	Canada	SibNIISKHOZ[c] 6	East Europe	Russia
Maplearrow	North America	Canada	PEP26	East Europe	Russia
Mapleglen	North America	Canada	Belor	East Europe	Russia
Mapleamber	North America	Canada	Soer345	East Europe	Russia
Gaillard	North America	Canada	Gribskaya	East Europe	Russia
KG 20	North America	Canada	Krasivaya mechta	East Europe	Russia
Accord	North America	Canada	Carola	North America	USA
AC Brant	North America	Canada	Daksoy	North America	USA
Kharbin	East Asia	China	Dawson	North America	USA
Kheikhek14	East Asia	China	USKHI[d] 6	East Europe	Ukrain
LMF	East Europe	Poland	Prikarpatskaya81	East Europe	Ukrain
Chabem Wekoju	East Europe	Poland	Chernovickaya7	East Europe	Ukrain
Arctic	East Europe	Poland	Spritna	East Europe	Ukrain
Kollekcyina	East Europe	Poland	Terek	East Europe	Ukrain
Nawiko	East Europe	Poland	Ustya	East Europe	Ukrain
Warsawska	East Europe	Poland	Khorol	East Europe	Ukrain
Kasatka	East Europe	Russia	Yug30	East Europe	Ukrain
Severnaya5	East Europe	Russia	Estophita	East Europe	Ukrain
Smena	East Europe	Russia	Podyaka	East Europe	Ukrain
Rassvet	East Europe	Russia	Victorina	East Europe	Ukrain
Amurskaya401	East Europe	Russia	Annushka	East Europe	Ukrain
Soer3491	East Europe	Russia	Amour	West Europe	France
Omskaya4	East Europe	Russia	Kalmit	West Europe	France
Soer-3	East Europe	Russia	Sepia	West Europe	France
Bryanskaya	East Europe	Russia	Amphor	West Europe	France
Nadejda	East Europe	Russia	Toury	West Europe	Czech Republic
Lidiya	East Europe	Russia	Rana	West Europe	Czech Republic
VNIIS[a] 1	East Europe	Russia	Turijskaja	West Europe	Czech Republic
Luchezarnaya	East Europe	Russia	Fiskeby5	West Europe	Sweden
PEP27	East Europe	Russia	Fiskeby3	West Europe	Sweden
Sibniik[b] 315	East Europe	Russia	Oyachi2	East Asia	Japan
VNIIS[a] 2	East Europe	Russia	308/1	Kazakhstan	Kazakhstan, Kostanay
Soer4	East Europe	Russia	422/1	Kazakhstan	Kazakhstan, Kostanay

Bara	East Europe	Russia	186/1	Kazakhstan	Kazakhstan, Kostanay
Zolotistaya	East Europe	Russia	173/1	Kazakhstan	Kazakhstan, Kostanay
Zlata	East Europe	Russia	126/1	Kazakhstan	Kazakhstan, Kostanay
Mageva	East Europe	Russia	209/1	Kazakhstan	Kazakhstan, Kostanay
Soer-5	East Europe	Russia	261/1	Kazakhstan	Kazakhstan, Kostanay
Okskaya	East Europe	Russia	350/1	Kazakhstan	Kazakhstan, Kostanay
Svetlaya	East Europe	Russia	362/2	Kazakhstan	Kazakhstan, East Kazakhstan Region
Maleta	East Europe	Russia	371/2	Kazakhstan	Kazakhstan, East Kazakhstan Region
Vega	East Europe	Russia	407/2	Kazakhstan	Kazakhstan, East Kazakhstan Region
Luchnadezhdy	East Europe	Russia	404/2 (Birlik KV)	Kazakhstan	Kazakhstan, East Kazakhstan Region
Sonata	East Europe	Russia	370/2	Kazakhstan	Kazakhstan, East Kazakhstan Region
Zakat	East Europe	Russia	Zara	Kazakhstan	Kazakhstan, Almaty
Svapa	East Europe	Russia	Roza	Kazakhstan	Kazakhstan, Almaty
Lancetnaya	East Europe	Russia	Misula	Kazakhstan	Kazakhstan, Almaty
Zernica	East Europe	Russia	Almaty	Kazakhstan	Kazakhstan, Almaty
Niva70	East Europe	Russia	Zhalpaksai	Kazakhstan	Kazakhstan, Almaty
Sibiryachka	East Europe	Russia			

a – All-Russian Scientific Research Institute of Agriculture
b – Siberian Scientific Research Institute of Feed Production
c – Siberian Scientific Research Institute of Agriculture
d – Ural Agricultural Institute
